# Implementation of an automated transition readiness assessment in a pediatric rheumatology clinic

**DOI:** 10.3389/fped.2024.1457651

**Published:** 2024-10-17

**Authors:** Melissa Argraves, Elizabeth Murray, Alysha Taxter, Kelly Wise, Paul T. Jensen, Alana Goldstein-Leever, Bethanne Thomas, Alexa Scott, James Gallup, Ashlee Leone, Stacy P. Ardoin, Vidya Sivaraman

**Affiliations:** ^1^ Division of Pediatric Rheumatology, Nationwide Children’s Hospital, Columbus, OH, United States; ^2^ Pediatric Residency Program, Nationwide Children’s Hospital, Columbus, OH, United States; ^3^ Division of Clinical Informatics, Nationwide Children’s Hospital, Columbus, OH, United States; ^4^ Department of Pharmacy, Nationwide Children’s Hospital, Columbus, OH, United States; ^5^ Intermountain Saint George Rheumatology, Intermountain Heath, Saint George, UT, United States; ^6^ Division of Pediatric Psychology and Neuropsychology, Nationwide Children’s Hospital, Columbus, OH, United States; ^7^ Center for Clinical Excellence, Nationwide Children’s Hospital, Columbus, OH, United States

**Keywords:** pediatric rheumatology, transition of care, transition readiness, quality improvement, clinical informatics, adolescents and young adults, pediatric rheumatic disease

## Abstract

**Background:**

Failure of successful transition to adult care for adolescents and young adults with chronic rheumatic diseases negatively impacts their health and wellbeing. Transition of care is a vital and complex process within pediatric rheumatology that can be difficult to execute. Use of quality improvement (QI) and clinical informatics (CI) can help implement transition programs.

**Local problem:**

Despite efforts to improve transition of care within our pediatric rheumatology clinic, it has been difficult to implement and sustain good transition practices including assessment of transition readiness. Using QI methodology and CI, this study aimed to improve transition readiness assessment from 12 to 30% and sustain for one year by surveying transitioning patients yearly.

**Methods:**

A transition-focused QI team utilized methods endorsed by the Institute for Healthcare Improvement and leveraged CI to improve survey completion. Control charts of survey completion rates were tracked monthly. Descriptive statistics were used to analyze survey responses.

**Interventions:**

Interventions focused on automation of patient surveys at regularly scheduled clinic visits.

**Results:**

1,265 questionnaires were administered to 1,158 distinct patients. Survey completion rose from a baseline of 12% to greater than 90% and was sustained over 18 months. Identified educational needs included health insurance, scheduling appointments, obtaining care outside of rheumatology clinic business hours, Electronic Health Record messaging, and refilling medications.

**Conclusions:**

By leveraging CI and QI methodology, we were able to assess transition readiness in more than 90% of our patients and identify gaps in self-management. Process automation can create sustainable transition practices.

## Introduction

Transition from pediatric to adult rheumatology is a necessary process for many of our patients given that rheumatic diseases are often life-long chronic illnesses. Active disease and adverse outcomes around the time of transfer are present in a significant percentage of patients (1, 2) and some of these patients ultimately do not end up under the care of adult rheumatologists. Successful transfer of care rates can be as low as 50% (3, 4). Patients who do not transition successfully and those with certain rheumatic diseases are at increased risk of mortality and poor health outcomes (1, 5). The transition process is complex and many pediatric rheumatology clinics struggle to effectively prepare adolescents and young adults (AYA) (6). Recognition of the need for strategic transition planning has been established for the past few decades and multiple societies including the American Academy of Pediatrics (AAP), the American Academy of Family Physicians (AAFP), the American College of Physicians (ACP), the European League Against Rheumatism (EULAR) and the Pediatric Rheumatology European Society (PRES) have guidelines surrounding transition of care (7, 8). Many of these guidelines utilize the GotTransition™approach which focuses on six core elements to transition (7). These six core elements include: development and dissemination of a transition policy; tracking and monitoring of transitioning patients; recurrent assessment of transition readiness skills and education to advance these skills; development of a healthcare transition plan with appropriate documentation for individual patients; transfer of care of a patient to adult practice; and finally, confirmation of transfer of care with an opportunity to solicit feedback from the patient. These six elements of transition have also been favorably viewed by AYA with rheumatic diseases. In focus groups, AYA reviewing these six elements reacted favorably to them and emphasized the importance of focusing on autonomy and independence to empower patients to advocate for themselves (9).

Transition of care has been *a priori*ty at our center for many years, but due to a busy and constantly evolving health system and team, it has been challenging to implement and sustain progress (10, 11). Finding ways to seamlessly integrate effective processes into practice is imperative. Assessing patient readiness to transition via a survey is a crucial step toward providing education on the skills needed for successful transition. These modifiable behaviors are deemed important for successful transition (12). The results of the transition readiness surveys are intended to be used to focus on education and self-management skills empowering our patients to gain autonomy in the healthcare system.

Using methods endorsed by the Institute for Healthcare Improvement (IHI) model for improvement (13), a multidisciplinary team within the Division of Rheumatology sought to improve transition by focusing on the six core elements for transition (3). Utilizing a systematic approach, different elements were targeted at various times. After successfully implementing the transition policy, the team shifted focus to address transition readiness. In January 2021, the transition quality improvement (QI) team created a new project aim with the goal of increasing yearly transition readiness assessment from 12 to 30% by December 2021 and sustaining for one year.

## Methods

### Context

**Figure 1:**
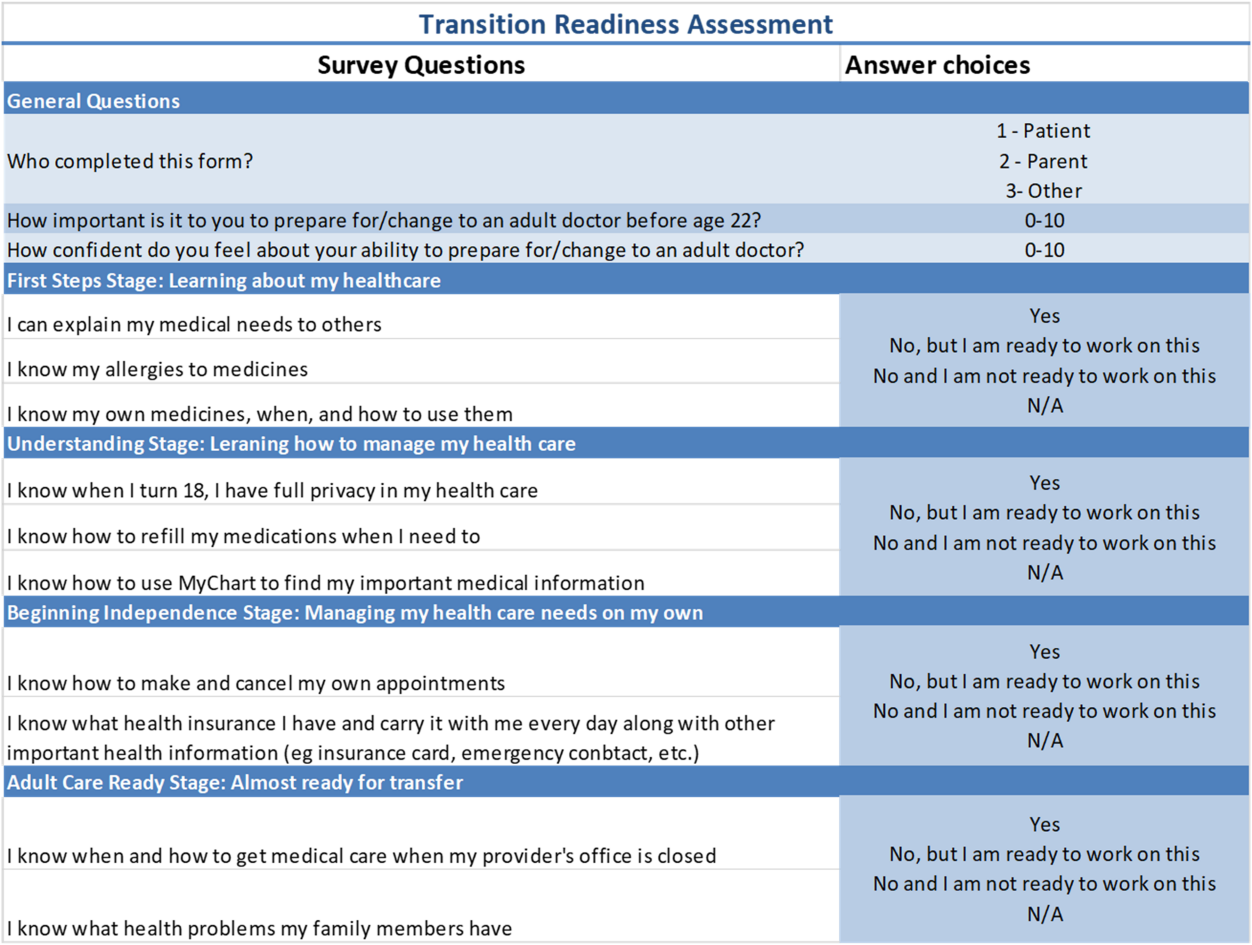
The questionnaire is 13 questions. The first column shows the questions, and the second column shows the potential answer choices. Please note that for questions 4-13, each question's answer choice is the same and includes the following options: yes; no, but I am ready to work on this; no, and I am not ready to work on this; N/A.

Nationwide Children's Hospital is a large, quaternary care children's hospital in Columbus, Ohio. The Division of Pediatric Rheumatology sees more than 1,600 unique patients annually. It is staffed by 8 pediatric rheumatologists, 1 adult and pediatric rheumatologist, 1 nurse practitioner (NP), 4 pediatric rheumatology fellow physicians, 1 psychologist, 1 clinical pharmacist, 1 social worker, and 7–10 part-time rheumatology nurses. One of the rheumatologists is a dual trained physician informaticist who joined the group in 2021. A transition QI team has been in place for more than 15 years. The team includes representatives from the rheumatology providers and allied health professionals listed above. As recommended by GotTransition™ (7), we defined patients aged 14 or older and seen at least 3 times in the rheumatology clinic as the target population of transitioning teens with rheumatic disease. A written transition policy was created in 2017. AYA receive a paper copy of the transition policy during the rooming process once they have reached the target population. Transition Readiness Assessment Questionnaires (14) were in use since 2011, but their usage was not consistent. In 2018, the QI team created a simplified transition readiness assessment in alignment with a hospital-wide initiative. The simplified questionnaire was given to the target population yearly, which we defined as every 12 months. This frequency was chosen to reduce survey burden and allow opportunity for responsive education at multiple time points. The transition readiness assessment consisting of 12 questions deemed critical by our transition QI team (Figure 1) was utilized. The questionnaire was available in the electronic health record (EHR) in English for patient completion during office visits beginning in July 2020 during the baseline data collection period. The survey was completed inconsistently by providers, social work, pharmacy, and the NP during transition teaching.

### Intervention

**Figure 2:**
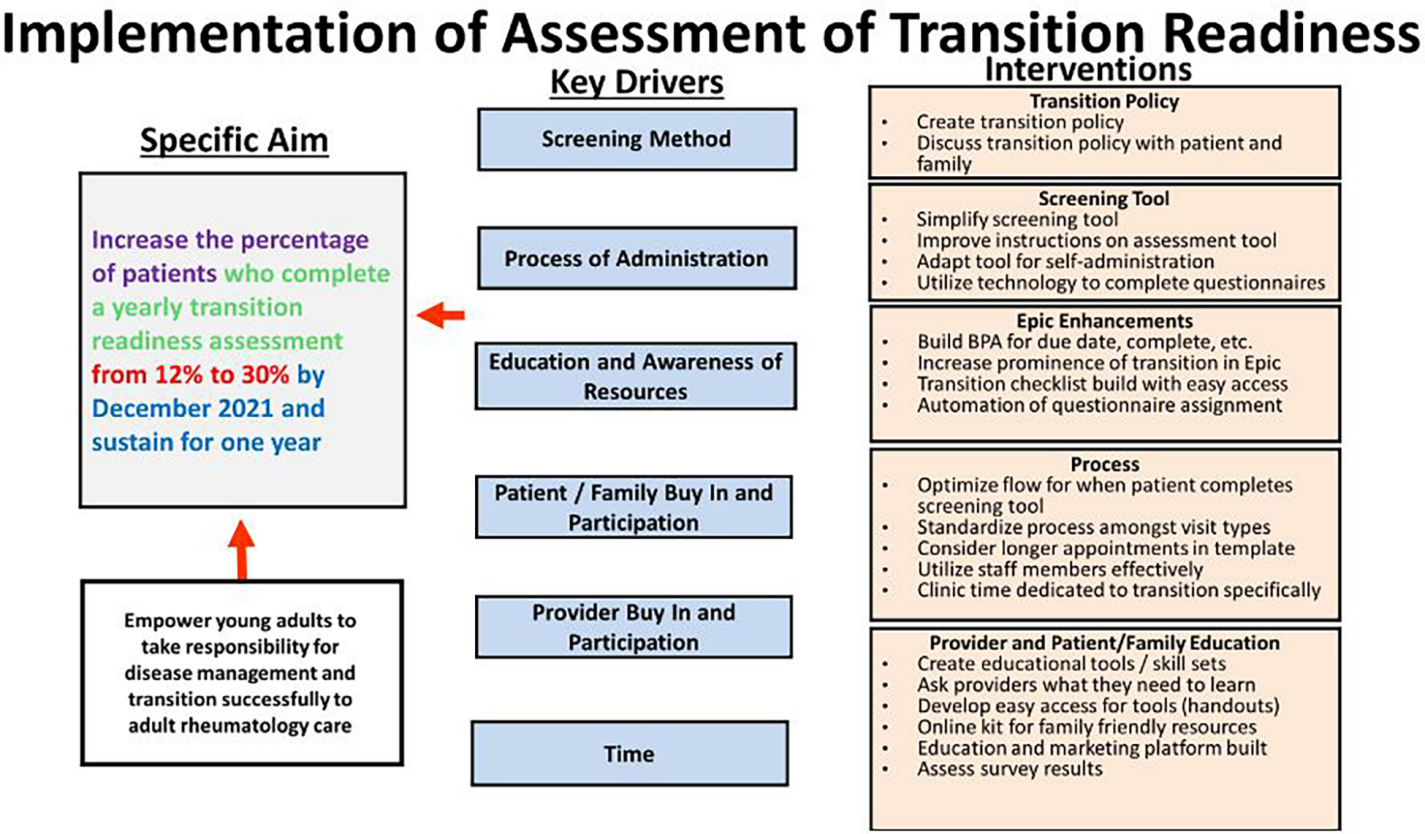
Key driver diagram.

Improvement of transition readiness assessment began in January 2021. A key driver diagram (Figure 2) was created to identify interventions to improve transition readiness assessment completion. Key drivers identified included the screening method, the process of administration, provider buy-in, patient and family buy-in and participation, education and awareness of resources, and time. Two Plan Do Study Act (PDSA) cycles occurred to improve questionnaire completion which utilized technology. In the first PDSA cycle, the questionnaire was automatically deployed on electronic tablets and given to appropriate patients at visit check in. The intervention leveraged existing practices with other patient surveys being completed electronically. The process required a clinic staff member to assign the questionnaire and provide the patient with the electronic tablet. In the second PDSA cycle, the questionnaires were automatically assigned to the patients with other intake questionnaires. They could be completed via the EHR patient portal prior to the visit with other questionnaires or were automatically loaded onto the tablets which all patients receive at check-in for a visit. We were able to achieve this by working closely with our hospital's informatics team and utilizing our dual trained informatics physician as a liaison. She put in the EHR build request via online form for the pre-existing survey to be loaded automatically onto the tablets. The survey had already been built in the EHR during the hospital wide initiative the year prior.

### Study of interventions

The percentage of patients with transition readiness assessments completed was measured each month in a statistical process control chart. Partial responses were counted. The transition QI team met monthly to review data and plan future interventions. A QI specialist assured the quality of the data and validated reports received from the hospital informatics team.

### Measures

Our main outcome measure as stated above was the percentage of patients with transition readiness assessments completed each month. Specifically, our numerator was the number of patients completing the transition readiness assessment each month and our denominator was the number of patients aged 14 and up who had been seen at least 3 times in rheumatology clinic and had a visit during that month. Additional process measures included percentage of questionnaires completed by patients (vs. parents/caregivers), and beginning in March 2023, percentage of questionnaires with all questions answered (i.e., tracking partial vs. complete survey completion). A balancing measure included the percentage of patients > age 21 seen in the past year to monitor if questionnaire completion and focus on transition readiness caused patients to transition later. While reviewing our data later in the stages of the project, it was noted that an incorrect denominator was being used that did not consider specific visit types including some of our specialty clinics, multidisciplinary clinics, and video visits. Therefore, the data was retroactively corrected beginning in April 2022 to add these visit types and more accurately represent our transitioning population. We also realized that the previous metric analyzing the percentage of patients who completed the assessment of all patients in the target population was flawed as this did not factor in the EHR automation that only prompted patients to complete the assessment once every 12 months. Following these discoveries, we reformatted our metric and control chart with the numerator defined as the number of patients who completed the transition readiness assessment in the last 12 months and the denominator as all patients 14 and older seen in rheumatology clinic at least 3 times for specified visit types within a given month. The entire division was updated on the project's progress and familiarized with where to access survey results within the EHR 1–2 times per year.

### Analysis

Questionnaire completion rates were collected from June 2019 to September 2023 with the baseline data period occurring from June 2019 to January 2021. A control chart was used to track progress of measures and evaluate the impact of interventions over time. In September 2023, we began to assess the quality of our patients’ survey responses to detect self-identified transition knowledge gaps at a population level by reviewing data collected from April 2022-September 2023. Using descriptive statistics, we looked at the percentage of questionnaires completed by patients vs. caregivers as well as self-management gaps. The self-management gaps were organized into a pareto chart to identify the most common gaps.

### Ethical considerations

Per institutional policy, this QI project was not considered human-subjects research. An Institutional Review Board (IRB) waiver was obtained.

## Results

**Figure 3:**
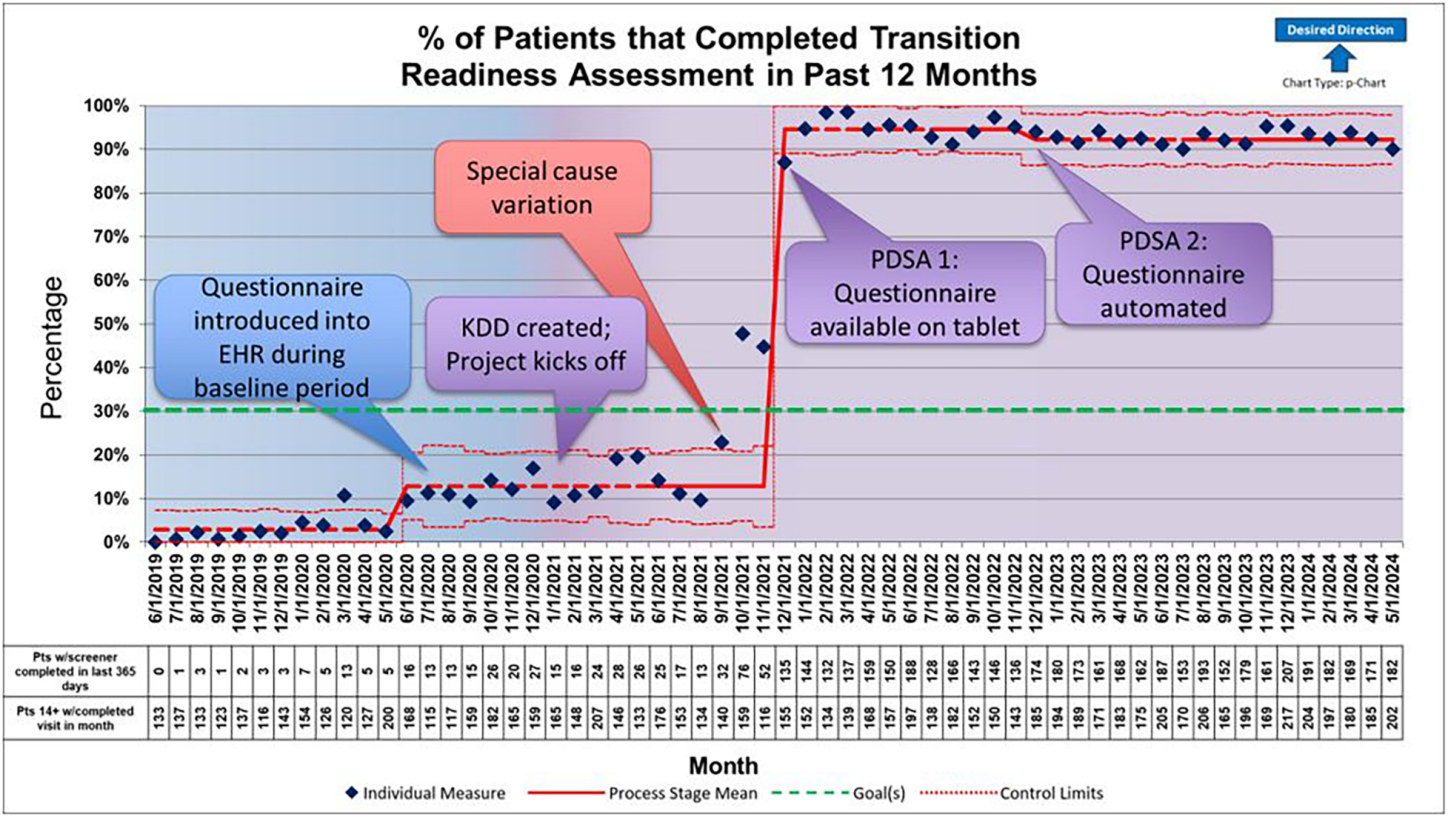
This control chart shows the percentage of patients with a completed transition readiness assessment in the past 12 months reported monthly from June 2019 until May 2024. Baseline data collection shown in blue and active quality improvement initiative shown in purple beginning in January 2021. The first Plan Do Act Study (PDSA) cycle occured in December 2021 and the second PDSA cycle occured in December 2022. Special cause variation was seen in September 2021 with a centerline shift in December 2021 after data points stabilized to a mean of 95%. It decreased slightly to 92% at the time of our second PDSA cycle in December 2022 and was sustained. Special cause variation was also seen in June 2020 during baseline data collection period.

**Figure 4:**
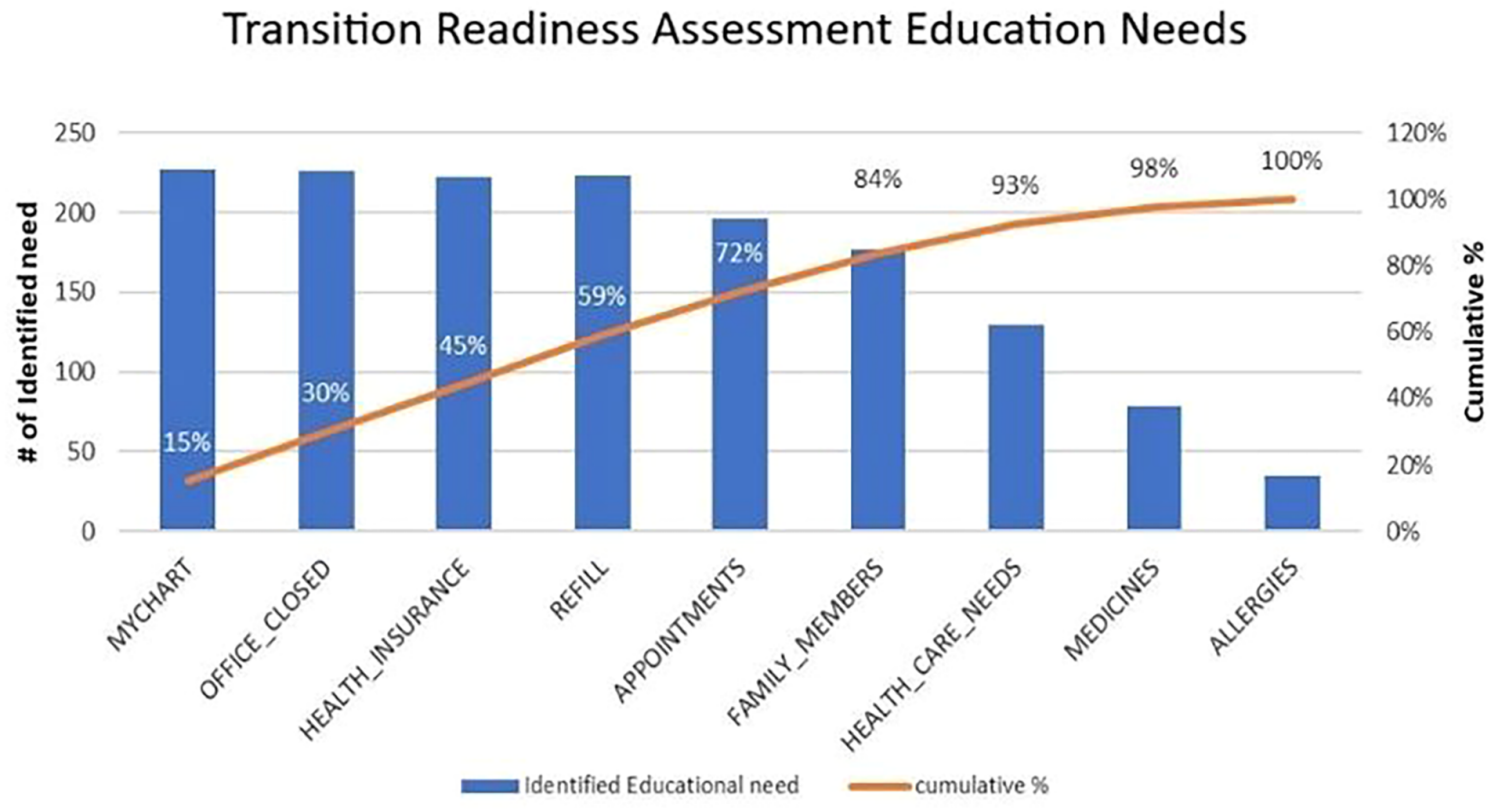
This pareto chart represents the most identified edicational needs based on data from the transition readiness assessment responses. Knowledge of patient portal use, what to do when the office is closed, knowledge of personal health insurance, understanding how to refill medication and kowning how to schedule and cancel appointments accounted for more than 80% of identified needs.

In total, 1,265 questionnaires were administered to 1,158 distinct patients; 74% were female and 97% spoke English as their primary language. Our baseline percentage of patients with transition readiness questionnaires completed was 12% following introduction of the questionnaire into the EHR. This event, while during the baseline data collection period, did result in special cause variation; the baseline percentage of patients with transition readiness questionnaires prior to this was 3%. Special cause variation began to occur in September 2021, prior to our first PDSA cycle, however the data continued to rapidly change so the center line did not shift until December 2021 correlating with our first PDSA cycle. The percentage of patients with transition readiness questionnaires completed rose to 95% initially with a slight decline to 92% in December 2022. As this shift occurred, our second intervention, automation of the questionnaire, was implemented and our center line remained steady (Figure 3). This completion rate was sustained for 18 months. The median age of patients completing the survey was 17 years old. Fewer than half of 14-year-old patients completed the questionnaire themselves; the patient completion rate increased to age 20–21, then decreased (Supplementary Figure S1). The most frequently identified educational needs were related to health insurance, scheduling appointments, obtaining care outside of rheumatology clinic business hours, EHR messaging, and refilling medications. These five factors accounted for more than 80% of the identified needs (Figure 4). Our balancing measure of percentage of patients greater than age 21 seen during each month decreased slightly from 3.5% to 3% during the project.

## Discussion

Using QI methodology and clinical informatics principles, we were able to successfully create a sustainable model for tracking transition readiness through automation of survey dissemination to our AYA patients yearly. We have achieved greater than 90% annual completion of transition readiness assessment. Our first PDSA cycle, electronic survey implementation, seemed to contribute most to the rise in completion rates though the special cause variation preceded the PDSA cycle by 3 months. This likely occurred due to increased focus on the questionnaire during this time. However, removing human factors such as the need for survey assignment improved sustained effect. The automation of the process is a strength of the project as it facilitates lasting sustainability (15).

Our findings are in alignment with other QI work within pediatric rheumatology which identify that automation within the EHR is an important facilitator of successful implementation of healthcare transition processes (16). This approach has allowed our efforts to sustain and collect data from most of our transitioning AYA. We will continue to use this approach as our efforts shift into the project's next phase. Several studies have looked at evaluating transition readiness with questionnaires, but few have taken a QI lens to improve implementation and only one has looked at questionnaire scores longitudinally (10). Interestingly, this study, which followed patient scores over time in a specialty clinic and did not have a formalized transition process, found that baseline scores did not predict transition or time to transition (10). It remains to be seen if addressing targetable skills will improve transition outcomes; however, self-efficacy, resilience, and patient activation have been shown to predict transition readiness scores suggesting that focusing on self-management skills and patient empowerment can improve scores and hopefully promote successful transition (17–20). While this is only one small step in a successful transition program, we believe that creating reliable assessment of transition readiness will allow future interventions to be more impactful.

As previously noted, our outcome measure was being inappropriately captured due to a false denominator. Fortunately, we have since adjusted this metric and our corrected completion rates are consistently greater than 90%. Additionally, we observed that a proportion of surveys were completed by parents, so results may inaccurately reflect the patients’ educational needs and transition readiness and instead reflect the parents’ perceptions. Our current process also does not distinguish between partial and total completion of questionnaires. We also do not have surveys for non-English speaking families which is a critical population that may be more vulnerable to unsuccessful transition.

We found it interesting that survey completion by patients increased only to age 21 and then decreased. However, when reflecting on the population of patients we serve greater than age 21, we note that many of them have intellectual disability. Many of them are unable to complete any questionnaire independently and thus parents are likely completing the questionnaires more in this age group.

While these issues are all current limitations of this project, we plan to address them in future iterations. Future directions will focus on improving the quality of the data and beginning to target educational interventions. To improve data quality, we plan to improve the percentage of questionnaires completed by patients, redefine survey completion to include all questions, measure incomplete survey completion as a process measure, and provide the survey to the most common non-English primary languages seen in our clinic. These changes should improve the utility of survey responses.

Survey responses are all viewable in our EHR in a specialty specific tab. Currently, there is no specific process in place to review these questionnaires with patients. It is up to the provider to review and discuss these responses and provide targeted education focused on improving patient knowledge and skill acquisition as needed. Our QI team is currently focusing on interventions to standardize this process. As a first step, we have started monitoring percentages of “yes” responses with a breakdown by age group for targeted and prioritized educational interventions. We will start by targeting the most desired educational needs including health insurance, scheduling, and medication refills identified in our pareto chart. The QI team just completed a brainstorming exercise and then created an effort/impact matrix to identify educational interventions and determine our next PDSA cycles.

Given the complexities of transition, QI efforts lend themselves to improving this challenging process. Even with a robust team and informatics support, this initiative took over 1 year to successfully implement and is only a small step in the overall goal of a comprehensive transition of care program. We hope this approach will encourage other centers to try to progress in their transition efforts. Our institution is fortunate to have many resources which help move this project along. However, even with more limited resources, utilizing a QI framework can help center transition efforts, and there are ongoing efforts to integrate more transition content into EHRs to facilitate this process. Based off our experience, we recommend the following:

• Start by formulating a QI transition team that meets at a regular interval. Measuring transition readiness can start out simply with paper survey administration.
• It is useful to leverage resources that institutions may have.
  ◦ If an institution has any QI team, utilize them as much as able, particularly for data collection.
  ◦ Find champions within the division, and utilize support staff such as nurses, social workers, and allied health professionals.
  ◦ Leverage existing practices in the division. If surveys are already being administered for other things, add a transition readiness assessment to that process.
  ◦ If automating or integrating into the EHR, utilize an informatics team.
• Meet regularly and expect delays. This process is not linear, and setbacks are normal and to be expected.

In conclusion, this initiative allowed us to identify modifiable knowledge gaps deemed necessary for successful transition on both a population and individual patient level. We will target these areas for knowledge acquisition in the next phase of our larger transition of care QI initiative.

## Data Availability

The raw data supporting the conclusions of this article will be made available by the authors, without undue reservation.
