# Supplementary figures and images for: Implementation of an automated transition readiness assessment in a pediatric rheumatology clinic
Source: Front Pediatr. 2024 Oct 17;12:1457651. doi: 10.3389/fped.2024.1457651 (PMC11524873; doi:10.3389/fped.2024.1457651)

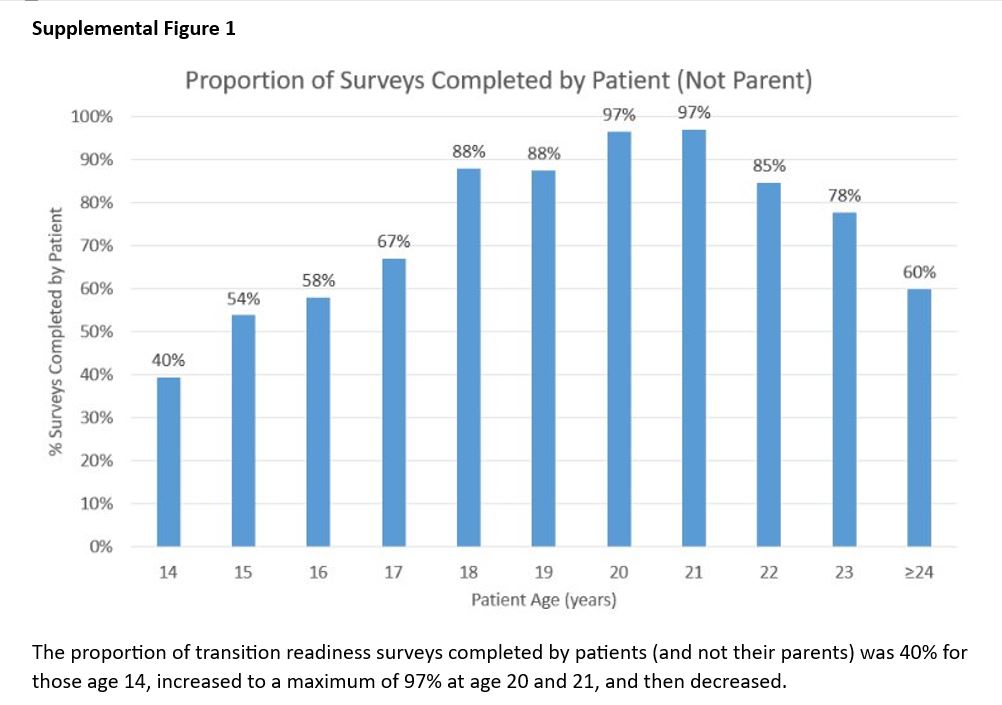

Supplement: Supplementary file 1 [file Image1.jpeg]
